# Transferability of Exercise Intensity Based on Muscle Oxygenation from Normoxia to Hypoxia in Ski-Mountaineering Athletes—Exploratory Study

**DOI:** 10.3390/sports12120351

**Published:** 2024-12-18

**Authors:** Kinga Rębiś, Tomasz Kowalski, Kamil Michalik, Andrzej Klusiewicz

**Affiliations:** 1 Department of Physiology, Institute of Sport—National Research Institute, 01-982 Warsaw, Poland; 2 Faculty of Physical Education and Sport, Wroclaw University of Health and Sport Sciences, 51-612 Wroclaw, Poland; 3 Department of Physical Education and Health in Biala Podlaska, Faculty in Biała Podlaska, Jozef Pilsudski University of Physical Education, 00-968 Warsaw, Poland

**Keywords:** near-infrared spectroscopy, NIRS, hypoxia, exercise testing, anaerobic threshold, SKIMO

## Abstract

Frequent changes in altitude and oxygen levels limit the practical application of traditionally derived exercise thresholds or training zones based on heart rate (HR) or blood lactate concentration (bLa). We investigated the transferability of a muscle oxygenation (SmO_2_)-based intensity prescription between different hypoxic conditions to assess the suitability of real-time SmO_2_ measurements for ski-mountaineering (SKIMO) athletes during submaximal endurance exercise. A group of 15 well-trained male SKIMO athletes performed a graded-intensity run test in normoxia (87 m ASL, FiO_2_ = 20.8%) to determine the anaerobic threshold (AnT) with the mod-Dmax method, and maximal lactate steady state (MLSS) assessments in acute normobaric hypoxia (3000 m ASL, FiO_2_ = 14.4%) with the intensity aligned to 90–105% of SmO_2_ at the normoxia-determined AnT. SmO_2_, HR, and bLa were monitored during both tests. The number of MLSS assessments without a bLa increase over 1 mmol·L^−1^ was reported. Paired *t*-tests with Cohen’s d effect sizes and intraclass correlation coefficient (ICC) were computed to compare the bLa and HR at the AnT in normoxia and MLSS averages in hypoxia, as both corresponded to equivalent SmO_2_. Out of the 15 MLSS assessments, 11 (73.3%) were performed without a bLa increase over 1 mmol·L^−1^. Significant differences at equivalent SmO_2_ in normoxia and hypoxia were found for HR (175 ± 11.7 vs. 160 ± 14.2 bpm, *p* = 0.005, d = 1.02), but not for bLa (4.9 ± 1.2 vs. 5.1 ± 2.4 mmol·L^−1^, *p* = 0.845, d = −0.05). ICC(2,k) for HR and bLa were 0.56 (95% CI: −0.24, 0.85) and 0.40 (95% CI: −0.75, 0.80), respectively. The results indicate a fair transferability of a SmO_2_-based intensity prescription between different hypoxic conditions in well-trained SKIMO athletes during submaximal endurance exercise. The practical significance of the observations depends on the required accuracy of the exercise intensity determination.

## 1. Introduction

Ski-mountaineering (SKIMO) is an endurance winter sport that combines skiing and mountaineering. As the participants ascend and descend snow-covered mountains, continuous effort in various hypoxic conditions is required. It is a rapidly growing discipline worldwide and has been included in the program for the Winter Olympic Games starting in 2026 [1,2]. Physiologically, the effort involved in SKIMO can be compared to cycling time-trial on a hilly route, running a 10 km race, or cross-country skiing. Therefore, SKIMO is one of the most physically demanding sports [3]. SKIMO may be described as a technical discipline with a significant endurance component, engaging both anaerobic and aerobic energy systems to varying degrees [4,5]. As a result, due to the mountain environment, altitude, and the significant role of technique in both climbing and descending, it is a discipline where assessing physical performance and monitoring training is particularly challenging [1]. Another factor complicating the analysis of training loads and the determination of training intensity zones is that a competition takes place in high mountains, with a variable hypoxic component. In a single race, participants may climb over 4000 m in elevation, repeatedly ascending above 3000 m above sea level [6]. A literature review shows a need for more detailed analyses, especially among elite SKIMO athletes, which is why new solutions are still being sought that could serve as tools to support the training process [1,3]. The specificity of SKIMO is linked to competitions being held at altitudes above 2000 m above sea level. Another important factor is the altitude change during the competition—athletes often move multiple times between zones ranging from 2000 to 3000 m above sea level, up to zones above 3000 m [6]. The complex physiological demands of SKIMO create a methodological challenge in selecting the most appropriate performance indicators to describe race intensity [3].

Traditionally, training zones and exercise thresholds are usually determined by heart rate (HR), pace, or blood lactate concentration (bLa) [7]. However, these indices may exhibit different values during exercise in hypoxia compared to at sea level [8,9,10]. In hypoxic conditions, oxygen uptake is typically reduced during both submaximal and maximal exercise due to the diminished partial pressure of oxygen, which limits oxygen availability for muscular work [11]. This reduction usually impairs exercise performance and leads to an earlier onset of fatigue [12]. To compensate, heart rate increases significantly at a given workload compared to normoxia, as the cardiovascular system attempts to maintain oxygen delivery to active muscles [11]. Furthermore, at a corresponding workload bLa may be higher in moderate hypoxia, reflecting an increased reliance on anaerobic glycolysis to meet energy demands in the face of limited oxygen availability during submaximal intensity. These physiological changes highlight the body’s adaptive responses to maintain energy production under hypoxic stress [13]. At the same time, adapted and trained individuals under high hypoxia may present a lower peak bLa, especially in association with high-intensity exercise [14].

These physiological changes may affect the comparability of laboratory measurements obtained under normoxic conditions, limiting their real-world applicability in the field. Such limitations are particularly noteworthy in disciplines that require performing in constantly changing conditions, such as SKIMO in the context of variable altitude and exercise intensity. Therefore, alternative methods of assessing exercise intensity and prescribing training are being sought.

As the traditional methods for assessing SKIMO performance and exercise intensity exhibit significant limitations, new approaches are still being explored. One of the modern tools that has gained popularity in training practice in recent years is mobile measurement devices utilizing near-infrared spectroscopy (NIRS). This technology allows for monitoring local muscle oxygenation saturation (SmO_2_) [15]. It was successfully applied in both normoxia and hypoxia in state-of-the-art research [16,17,18]. The reports on the SmO_2_ kinetics in hypoxia during exercise are scarce and not conclusive. For example, Solsona et al. (2022) observed that SmO_2_ might be similar across different hypoxic conditions with a decrease in total work [19] during sprint interval training. Pramkratok et al. (2022) also reported stable SmO_2_ levels during incremental running tests with impaired performance in hypoxia [20]. On the contrary, Rodriguez et al. (2019) noted that locomotor muscle oxygenation was reduced, whilst oxygenation of respiratory muscles was maintained in hypoxia during repeated sprint efforts [21]. Noteworthy, both the applied exercise types and the population in the available research differ vastly. It may suggest that the SmO_2_ kinetics depend on the exercise intensity, modality, participants’ fitness level, or applied research methodology. Consequently, further and more specific research is required. According to the literature data and recent investigations, NIRS measurements allow for both assessing maximal exercise capacity and determining training intensity zones from an incremental exercise test [22,23]. Furthermore, analyzing the dynamics of SmO_2_ changes during exercise enables the determination of individualized training zones and intensity thresholds [23]. Noteworthy, the available research focuses on measurements performed in the same conditions [24,25], and the application of such an approach during endurance efforts in various hypoxic conditions remains understudied. This might be one of the first investigations addressing such an application of NIRS in outdoor winter sports, conducted on well-trained athletes.

We hypothesized that NIRS-based measurements may optimize for the transfer of laboratory test results, conducted in normoxia, to hypoxic conditions. Additionally, we speculated that the proposed training zones should remain stable regardless of altitude, unlike traditional training zones based on HR, pace, or bLa. Therefore, we investigated the transferability of SmO_2_-based intensity prescription between different hypoxic conditions to assess the suitability of real-time SmO_2_ measurements for SKIMO athletes during submaximal endurance exercise.

## 2. Materials and Methods

The study design was reviewed and approved by the Institute of Sport—National Research Institute Ethics Committee (approval no KEBN-24-97-KR). All procedures are aligned with the Declaration of Helsinki. All study participants provided written consent to take part in the experiment.

### 2.1. Participants

A group of male SKIMO athletes (n = 15) completed the study. The participants were classified as either Tier 3 or Tier 4 athletes according to the participant classification framework [26], indicating they were trained or highly trained athletes. The inclusion criteria required participants to have a valid medical certificate for SKIMO training and competition, be classified in Tier 3 or Tier 4 as defined by McKay et al. (2002), and have at least 4 years of competitive athletic training. Exclusion criteria included any chronic or acute medical conditions within the past 3 months and the current use of medication. The recruitment was based on convenience sampling through the Polish National Team coaches. The required sample size was calculated with G* Power (version 3.1.9.2; Dusseldorf, Germany) and totaled 15 participants. The following input parameters were set as follows: effect size = 0.8, significance α = 0.05, and power (1 − β) = 0.8. Initially, 18 athletes enrolled, but only 15 completed all the necessary procedures and were included in the analyses. The participants’ characteristics are presented in Table 1.

The participants’ height was measured with the free-standing digital stadiometer seca 274 (seca GmbH & Co. KG, Hamburg, Germany). Their body mass was measured with the body composition analyzer InBody 770 (InBodyUSA, Cerritos, CA, USA). Maximum oxygen uptake (VO_2_max) was established during a graded-intensity run test. The gasometry was performed with the Cortex Metamax B3 (Cortex Biophysik GmbH, Leipzig, Germany), breath-by-breath method, according to the manufacturer’s guidelines. All the included participants fulfilled the maximal effort criteria: (1) HR ≤ 10 beats/min or ≤5% of the age-predicted (220 - age) maximum, (2) bLa ≥ 8 mmol/L, and (3) respiratory exchange ratio > 1.10 [27]. The highest average oxygen uptake for 30 s was considered as the VO_2_max.

### 2.2. Study Design

Across 10 days the athletes took part in two testing procedures to determine the transition between heavy and severe exercise domains. First, the participants performed a graded-intensity run test on a treadmill (h/p/cosmos Saturn treadmill, h/p/cosmos sports & medical GmbH, Nussdorf-Traunstein, Germany) in normoxia (87 m ASL, FiO_2_ = 20.8%). The test was conducted to exhaustion, with a 4 min step protocol and capillary blood sampling for bLa after each step. The anaerobic threshold (AnT) with the mod-Dmax method was established for HR, bLa, and SmO_2_ [28]. The following week, the participants performed maximal lactate steady state (MLSS) run assessments in acute normobaric hypoxia (3000 m ASL, FiO_2_ = 14.4%) with the intensity aligned to 90–105% of SmO_2_ at the normoxia-determined AnT. The 15% range for SmO_2_ reflected the constant acute changes of the measurement and resembled a training zone that may be practically used in training. The MLSS assessment was based on three 10 min efforts with a 1 min recovery interval. The speed and incline were self-adjusted by participants to keep the intensity in the prescribed SmO_2_ range. The average HR was measured during the last minute of each effort and the bLa was measured immediately after each effort. Hypoxic conditions in the laboratory were generated using the Airzone normobaric hypoxia system (Air Sport, Międzyzdroje, Poland), with the hypoxic chamber measuring ca 45 m^2^. Temperature, humidity, oxygen, and carbon dioxide concentrations were controlled and held constant.

SmO_2_ was measured with a Moxy monitor (Moxy Monitors, Hutchinson, MI, USA), previously validated and widely applied in sports settings [29]. The device was placed on the right vastus lateralis, about 13 ± 1 cm above the upper edge of the patella and 4 ± 1 cm to the side of the thigh’s midline. A nontransparent tape of 7.5 cm width was used to provide a repeatable testing environment and hypoallergenic skin tape was used to fix devices to the skin. A computation window of 2 s was used. Skinfold thickness at this location was measured using a Harpenden Skinfold Caliper (Baty International, Burgess Hill, United Kingdom). The recorded values (5.3 ± 1.3 mm) ensured physiologically reliable SmO_2_ measurements [30]. HR was measured with a Polar H10 chest strap (Polar Electro Oy, Helsinki, Finland). Blood samples were taken from the fingertip into 20 μL capillary tubes and immediately analyzed with a Super GL 2 (Dr. Müller Gerätebau GmbH, Freital, Germany) for the bLa.

### 2.3. Statistical Analysis

The number and percentage of MLSS assessments without a bLa increase over 1 mmol·L^−1^ was reported. The normality of the distribution was assessed with the Shapiro–Wilk test and visual analysis of the plot figures. Scatter plots were analyzed for independence of observations, linearity, and outliers. Paired *t*-tests with Cohen’s d effect sizes and intraclass correlation coefficient (ICC) were computed to compare bLa and HR at AnT in normoxia and MLSS averages in hypoxia, as both corresponded to equivalent SmO_2_. Bland–Altman plots were presented to visualize the agreement between both measurements. All statistical analyses and adequate figures were prepared using the JASP Team statistical package JASP (JASP Team, Amsterdam, The Netherlands, Version 0.17.2).

## 3. Results

**Figure 1:**
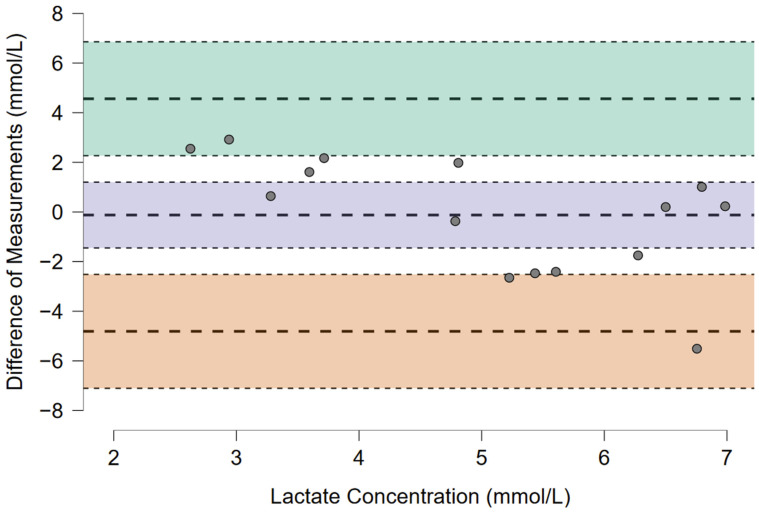
The Bland–Altman plot presents an agreement between the bLa at AnT in normoxia and the MLSS averages in hypoxia (both corresponded to equivalent SmO_2_).

**Figure 2:**
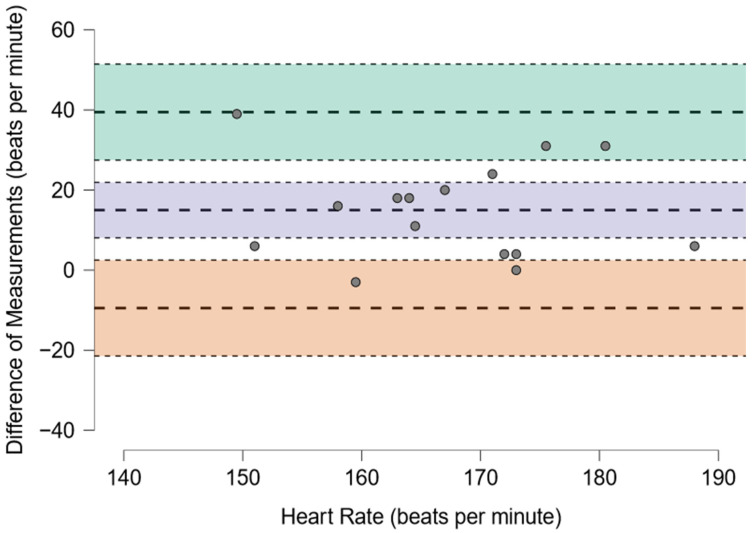
The Bland–Altman plot presents an agreement between the HR at AnT in normoxia and the MLSS averages in hypoxia (corresponded to equivalent SmO_2_).

Out of the 15 MLSS assessments, 11 (73.3%) were performed without a bLa increase over 1 mmol·L^−1^. Statistically significant differences at equivalent SmO_2_ in normoxia and hypoxia were found for the HR (175 ± 11.7 vs. 160 ± 14.2 bpm, *p* = 0.005, d = 1.02), but not for the bLa (4.9 ± 1.2 vs. 5.1 ± 2.4 mmol·L^−1^, *p* = 0.845, d = −0.05). ICC(2,k) for the HR and bLa were 0.56 (95% CI: −0.24, 0.85) and 0.40 (95% CI: −0.75, 0.80), respectively. The Bland–Altman plots to visualize the agreement between the measurements are presented as Figure 1 and Figure 2.

## 4. Discussion

Our study investigated the transferability of SmO_2_-based intensity prescription between different hypoxic conditions in well-trained SKIMO athletes during submaximal endurance exercise. This is a critical area of research as traditional methods for prescribing exercise intensity, such as HR, pace, or bLa, are known to be influenced by altitude, potentially leading to inaccurate exercise intensity prescriptions. To our knowledge, this is the first study exploring the application of NIRS-based indices in different conditions. The results indicate that SmO_2_-based intensity prescription demonstrates a fair transferability between normoxic and hypoxic conditions within intensities close to the transition between the heavy and severe exercise domains. Our findings suggest that SmO_2_, measured with portable NIRS devices, may offer a fairly stable and reliable method for setting training zones across varying altitudes during submaximal endurance exercise. This is crucial for optimizing training adaptations and racing strategies in SKIMO athletes who frequently train and compete at altitude.

Since the introduction of the equilibrium principle to lactate production and clearance [31], numerous studies have explored MLSS, using a variety of equipment, exercise modalities, movement patterns, muscle groups, and endurance performance measures [32]. Although the precise definition and interpretation of MLSS vary, many authors have referred to the MLSS as the AnT because it represents an exercise intensity that can be sustained without significant reliance on anaerobic metabolism [33]. Generally, a close relationship between traditional exercise thresholds and MLSS suggests validity concerning the training intensity prescription [33]. However, multiple scientists reported only a loose correlation between MLSS and bLa-derived exercise thresholds [34,35]. De Souza et al. (2012) reported moderate to high correlations (r = 0.68 and r = 0.79) between MLSS and aerobic or anaerobic thresholds, respectively [34]. Urhausen et al. (1993) reported that bLa increased continuously and/or led to a premature break-off during the ‘quasi-MLSS’ protocol in 15 out of 30 cases [35]. Aunola and Rusko (1992) reported that AnT and MLSS are correlated (r = 0.83); however, no correlation between the 4 mmol lactate threshold and MLSS was observed [36]. Interestingly, Smith and Jones (2001) found no significant differences between MLSS velocity and lactate turnpoint velocity but noted that the extent of disagreement is too great to estimate one variable accurately from another in individual subjects [37]. Hoogeveen et al. (1997) also reported the conspicuous individual variability of bLa at MLSS in well-trained endurance athletes [38]. During our investigation, 73.3% of MLSS assessments were performed without a bLa increase over 1 mmol·L^−1^ and no significant differences between the bLa in normoxia and hypoxia were found. Considering the presented context, the approach investigated in our study seems promising.

The practical significance of our observations depends on the needed accuracy of the exercise intensity determination. Typically, very high precision is required in performance-oriented environments [39], whereas applied population approaches to accuracy and repeatability are often less demanding [40,41]. Hence, fair transferability between normoxic and hypoxic conditions may be sufficient to play a role in limiting acute mountain sickness in the general tourist population at moderate altitudes, as high-intensity exercise may exaggerate arterial hypoxemia leading to a higher prevalence of negative altitude symptoms [42]. At the same time, fair transferability may seem not enough for world-class athletes and coaches, as precise and calculated training models are desired in high-performance environments [39].

### 4.1. Practical Implications for Endurance Exercise

Compared to bLa measurements, SmO_2_ monitoring offers several advantages for prescribing exercise intensity, such as real-time data availability. NIRS technology allows for non-invasive, continuous monitoring of SmO_2_ during exercise, providing real-time feedback on SmO_2_ dynamics that reflect exercise intensity [24]. State-of-the-art NIRS devices typically involve a small wearable sensor, that transmits data wirelessly to a sports watch or smartphone app [43]. This technology eliminates the need for cumbersome equipment and frequent blood sampling, making SmO_2_ monitoring practical and accessible for both laboratory and field-based assessments [44]. Commercial NIRS devices are portable and mobile, allowing for continuous data collection even in challenging outdoor environments like those encountered in SKIMO. The integration of these wearable devices with existing training platforms and software further enhances data analysis and interpretation, allowing for individualized training prescriptions and real-time feedback for athletes.

The practical implications of our findings are significant for SKIMO athletes and their coaches. By adopting a SmO_2_-based intensity prescription, they could potentially benefit from the following:

- Optimize training adaptations. SKIMO athletes could train at more precise and consistent intensities across different altitudes, maximizing physiological adaptations specific to SKIMO performance.
- Individualize training prescription. SKIMO athletes could utilize real-time SmO_2_ data to tailor training loads, techniques, and recovery strategies based on individual athlete responses to training and environmental conditions.
- Monitor training load and fatigue. SKIMO athletes could track changes in SmO_2_ over time to assess training adaptations, identify early signs of fatigue, and potentially reduce the risk of overtraining.

However, the proposed approach should be applied with caution, as high individual variability concerning NIRS-obtained indices has been reported [45]. Noteworthy, the presented approach might be successfully used in other sports disciplines that compete in different hypoxic conditions or rely on altitude training in their preparations.

### 4.2. Study Limitations and Future Research

The study design is associated with the exploratory character of our investigation and therefore is not free from limitations. First, the sample size was relatively small and included only male ski-mountaineers. Next, the compared variables have applied character and the direct juxtaposition of the same protocols in normoxia and hypoxia is lacking. Finally, the research addressed a homogeneous group of well-trained endurance athletes that regularly train and compete in hypoxia; therefore, our findings should not be extrapolated to different populations, i.e., recreationally active tourists. Consequently, future research might address efforts with different characteristics (i.e., long in duration and low in intensity) or groups of different genders and training statuses. Our study addressed the intensities close to the transition between heavy and severe exercise domains and should be validated with different work rates. Since substantial individual variability in analyzed indices was observed, it is important to explain the sources and relationships associated with described intra-subject divergence. Moreover, other modalities such as cycling or cross-country skiing should be explored since significant differences between exercise types might appear [46]. Furthermore, the findings should be translated into clear and actionable guidelines for coaches and athletes based on out-of-the-laboratory measurements. Preferably, the testing environment should reflect the training and racing conditions [47] to further optimize the implementation of SmO_2_-based training prescriptions.

## 5. Conclusions

Our findings indicate that a SmO_2_-based intensity prescription demonstrates a fair transferability between normoxic and hypoxic conditions during submaximal endurance exercise. While traditional methods remain relevant, incorporating SmO_2_ monitoring may provide a more nuanced and potentially more accurate approach to training prescription, particularly for individuals exercising, training, or competing in various hypoxic environments.

## Figures and Tables

**Table 1:** Baseline participant characteristics.

Variable	Value (Mean ± Standard Deviation)
Age (years)	29.7 ± 11.5
Body mass (kg)	71.4 ± 4.3
Body height (cm)	179.1 ± 7.5
VO_2_max (mL·min^−1^·kg^−1^) ^1^	60.9 ± 8.1
Body mass (kg)	71.4 ± 4.3

^1^ VO_2_max—maximal oxygen uptake.

## Data Availability

Data will be made available upon reasonable request to the corresponding author (K.R.).
